# A Deep Learning Method for the Impact Damage Segmentation of Curve-Shaped CFRP Specimens Inspected by Infrared Thermography

**DOI:** 10.3390/s21020395

**Published:** 2021-01-08

**Authors:** Ziang Wei, Henrique Fernandes, Hans-Georg Herrmann, Jose Ricardo Tarpani, Ahmad Osman

**Affiliations:** 1 Fraunhofer IZFP Institute for Nondestructive Testing, 66123 Saarbrucken, Germany; ziang.wei@izfp-extern.fraunhofer.de (Z.W.); hans-georg.herrmann@izfp.fraunhofer.de (H.-G.H.); 2 School of Engineering, University of Applied Sciences, 66117 Saarbrucken, Germany; 3 Faculty of Computing, Federal University of Uberlandia, Uberlandia 38408-100, Brazil; 4 Chair of Lightweight Systems, Saarland University, 66123 Saarbrucken, Germany; 5 Sao Carlos School of Engineering (EESC-USP), Sao Carlos 13566-590, Brazil; jrpan@sc.usp.br

**Keywords:** composite materials, infrared thermography, deep learning, damage segmentation, curve shaped laminates

## Abstract

Advanced materials such as continuous carbon fiber-reinforced thermoplastic (CFRP) laminates are commonly used in many industries, mainly because of their strength, stiffness to weight ratio, toughness, weldability, and repairability. Structural components working in harsh environments such as satellites are permanently exposed to some sort of damage during their lifetimes. To detect and characterize these damages, non-destructive testing and evaluation techniques are essential tools, especially for composite materials. In this study, artificial intelligence was applied in combination with infrared thermography to detected and segment impact damage on curved laminates that were previously submitted to a severe thermal stress cycles and subsequent ballistic impacts. Segmentation was performed on both mid-wave and long-wave infrared sequences obtained simultaneously during pulsed thermography experiments by means of a deep neural network. A deep neural network was trained for each wavelength. Both networks generated satisfactory results. The model trained with mid-wave images achieved an F1-score of 92.74% and the model trained with long-wave images achieved an F1-score of 87.39%.

## 1. Introduction

Composite materials (CM) are a class of advanced materials that are formed by the combination of two or more constituents. Usually they are formed by a matrix and a reinforcement. The combination of these two materials brings improved mechanical properties to the final assembly in terms of stiffness, strength, weight (lightness), high fatigue life flexibility, durability, and economical competitiveness [1,2,3]. Today, CM are used in the industry as replacements of other classical materials. They are widely used in transportation—including automotive, aeronautics and marine industries—architecture and civil infrastructure, renewable energy technology, and so on.

In the aerospace industry, structural components operating in harsh environments are now built with carbon fiber-reinforced thermoplastic (polyphenylene sulfide–PPS–C) matrix composite thin laminates (e.g., NASA’s Soil Moisture Active Passive—SMAP—satellite (https://smap.jpl.nasa.gov/)). For structural laminated composite materials, thermal and/or mechanical cyclic stresses followed by impact loading give rise to damages such as matrix cracking, fiber breaks, interply delamination, and fiber/matrix debonding. Low earth orbit (LEO) geostationary satellites can experience temperatures as low as −190 ${}^{\circ }C$ in the eclipse region (Earth’s shadow), and up to 150 ${}^{\circ }C$ in the sunlight region (i.e., temperature range approaching 300 ${}^{\circ }C$), so that repeated thermal shocks can still worsen the conditions faced by composite laminates permanently exposed to low-energy impact events by micrometeoroids [4,5,6]. Therefore, non-destructive testing and evaluation (NDT&E) techniques are essential to ensure structural safety, reliability, and operational life [7,8,9,10,11,12,13].

A large number of experimental techniques have been proposed in the last decades to assess the internal structure of CM. NDT&E can be grouped in different inspection categories, namely, visual inspections, imaging techniques, electromagnetic-field-based testing, acoustic-wave-based inspections, and optical techniques.

Among the most valuable NDT&E methods for the inspection of CM, infrared thermography (IRT) [14] holds a prominent position by providing fast surface inspection, easy deployment, safety, and relatively low cost. Thermal methods for NDT&E are based on the principle that heat flow in a material is altered by the presence of some types of anomalies [15]. Usually a heat pulse is used to start this heat flow. A heat pulse can be considered as the combination of several periodic waves at different frequencies and amplitudes. After the thermal front comes into contact with the specimen’s surface, a thermal front travels throughout the specimen. As time elapses, the surface temperature will decrease uniformly for a piece without internal flaws. On the contrary, subsurface discontinuities (porosity, delaminations, fiber breakage, etc.), can be considered as resistances to heat flow that produce abnormal temperature patterns at the surface, which can be detected with an infrared (IR) camera. The imaging or study of such thermal patterns is known as IRT.

IRT inspection of a structure usually produces a lot of data. Thousands of images can be acquired during one single experiment. These data must be processed to extract knowledge about the inner structures of composite components. With this regard, machine learning can play a key role in defect and damage assessment [16,17,18,19,20]. Deep learning is a machine learning approach that is based on neural networks. In principle, a deep and complex network allows multiple data processing steps aimed at the generation of consecutive representations of the inputs in feature spaces of increasing meaning. Besides, as deep models are always seen as "black box" because their lack of transparency, certain methods should be performed to reveal the data processing operations of the models.

In this paper, curved carbon fiber-reinforced thermoplastic matrix laminates are inspected by means of IRT. The specimens were submitted to different numbers of thermal-shock cycles and then impacted with a projectile with kinetic energy resembling that exhibited by micrometeorites. Each specimen was individually inspected using pulsed thermography (PT). IR raw sequences were then processed with a known IR processing method called principal component thermography (PCT) towards damage detection and characterization. Images obtained with PCT were used as ground truth for a supervised train of a convolutional neural network. Raw sequences with sample normalization operation were utilized as input for the network which segments impact damages present in the specimens.

The reminder of this paper is organized as follows: Section 2 describes the tested specimens, inspection technique and applied classification models. Results are presented in Section 3, followed by discussion in Section 4. Finally, conclusions and future work are provided in Section 5.

## 2. Materials and Methods

### 2.1. Inspected Specimens

Toray^TM^ (formerly TenCate^TM^) Cetex^TM^ TC1100 is a high-end cost-effective laminate reinforced with 5 HS, T300JB carbon fibre woven pre-impregnated with thermoplastic semi-crystalline polyphenylene sulfide polymer (hereafter PPS-C laminate) for excellent mechanical properties and outstanding chemical and solvent resistance. Six [0/90] plies were hot pressed for a 2 mm-thick, ${\left[0/90\right]}_{3S}$ laminate array. The final laminate is supplied with a thin glass fiber-reinforced PPS matrix top layer to protect a partly metallic assembly against galvanic corrosion. Basic properties are listed in Table 1 and Table 2.

**Figure 1:**
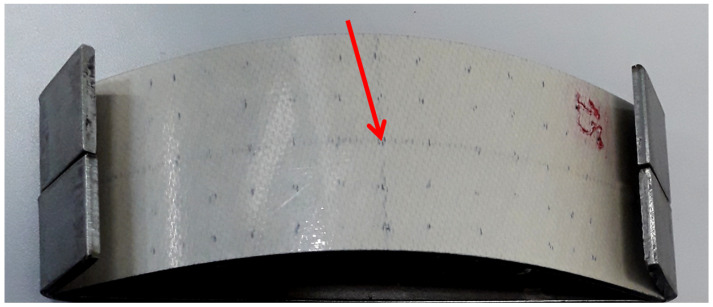
Schematic of the transverse impact test.

**Figure 2:**
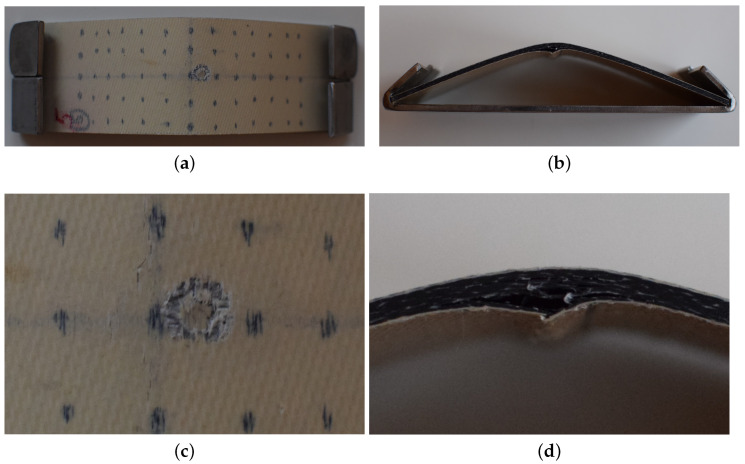
Curved specimen impacted after 300 thermal-shock cycles: (**a**) frontal view, (**b**) lateral view, (**c**) impact detail in the frontal view, and (**d**) impact detail in the lateral view.

Test specimens with in-plane dimensions of 150 × 45 mm were carefully machined from the PPS-C laminate and subsequently curved (curvature radius 120 mm) at ambient temperature. The curve-shaped specimens were permanently stabilized using metallic clamps and subjected to thermal shock cycles. For this purpose, they were repeatedly immersed in boiling water (100 ${}^{\circ }C$) and liquid nitrogen (−196 ${}^{\circ }C$), respectively. They remained immersed for 3 min in each liquid medium to guarantee thermal stabilization. Once they were retrieved from the boiled water contained, they were immediately transferred to the liquid nitrogen one, and vice-versa, to permanently warrant harsh thermal-shock conditions; 150, 300, and 500 thermal-shock cycles were applied to different specimens aiming at simulating thermal conditions experienced by geostationary satellites operating in low-earth orbit (LEO) environment. It is worth mentioning that some specimens were not heat-treated. Finally, the test coupons were transversely impacted (Figure 1) with air-driven (CBC^TM^ 5.5 Standard air rifle) caliber 5.5 mm cylindrical lead projectile weighting 1.6 g, traveling at a speed of 250 m/s under ambient temperature. Estimated impact energy was 50 J, which approaches typical energies for simulating micrometeoroid collisions [21] that may lead to severe damage to thin laminate composites. It is worth noticing that some test coupons were not impacted. A total of eight specimens were tested in this work. Figure 2 shows a curved specimen previously submitted to 300 thermal-shock cycles followed by impact. Table 3 lists all inspected specimens.

### 2.2. Infrared Thermography

NDT&E has been defined as comprising those methods used to examine or inspect a part, material, or system without impairing its future usefulness [14]. IRT and thermal methods for NDT&E are based on the principle that heat flow in a material is altered by the presence of some types of anomalies. These changes in heat flow cause localized temperature differences in the material surface. Some advantages of IRT are fast surface inspection, ease of deployment, and safety. In the active IRT approach, an external heat (or cold) source is used to produce a thermal contrast between the feature of interest and the background. There are some heat sources that could be used for this purpose. Here, an optical energy source was used.

**Figure 3:**
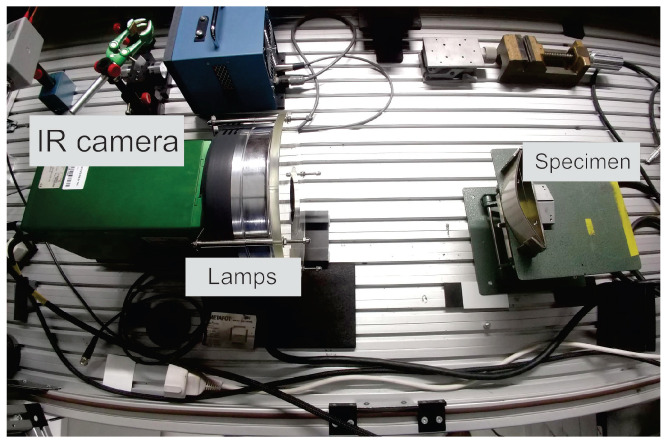
Reflection mode PT set-up used in this study.

To inspect the specimens, an IR camera equipped with a circular flash acting as an optical energy source was used, as shown in Figure 3. Both camera and optical energy source were placed in before the target, i.e., reflection mode, and a short flash pulse was triggered while the camera captured a sequence of images during some seconds. This inspection approach is called pulsed thermography (PT) [22]. A Thermosensorik QWIP Dualband 384 infrared dual-band camera was employed working simultaneously within the 4.4–5.2 μm (mid-wave infrared-MWIR) and 7.8–8.8 μm (long-wave infrared-LWIR) bands. A Xenon round flash with maximal 6 kJ was used. A 10 μs flash ( 3 kJ) was fired while the infrared camera started recording a 7 s long video at a frame rate of 150 fps, so that two raw sequences (respectively MWIR and LWIR ranges) were registered. The distance between the camera/flash and the inspected specimen was approximately 29 cm and the surfaces of the specimens were not damaged during the inspections. The entire set-up was surrounded by dark glass where the ambient temperature was 22.3 ${}^{\circ }C$ and ambient humidity around 42%. Emissivity of the inspected surface was considered to be around 0.95. All experiments were performed in the same day.

### 2.3. Thermal Data Analysis

**Figure 4:**
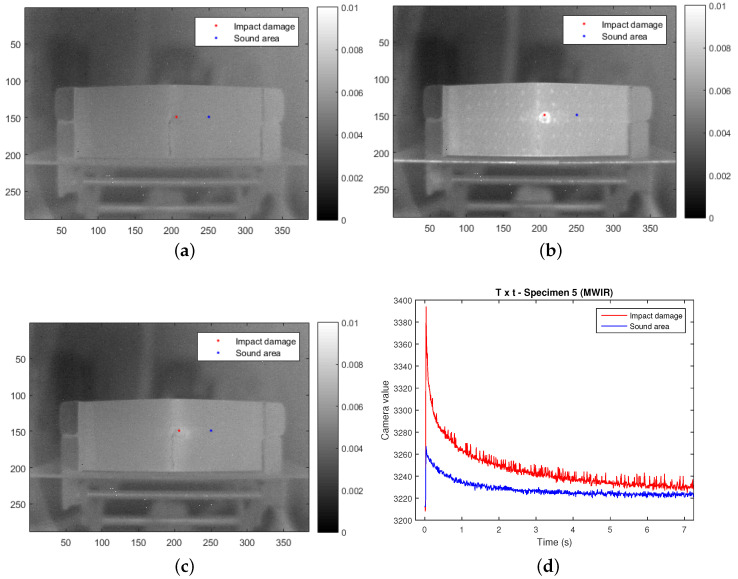
Raw MWIR data for specimen 5: (**a**) before flash pulse, (**b**) at 0.25 s, (**c**) at 3 s, and (**d**) temperature profiles of impacted (red) and sound (blue) regions (regions are also marked in the raw images).

MWIR and LWIR sequences having 1050 images of 288 × 384 pixels are available for each specimen. Examples of a MWIR sequence are provided in Figure 4. Figure 4a shows the raw cold image, i.e., the image before flash pulse; Figure 4b shows the image which corresponds to 0.25 s after the flash pulse; and Figure 4c shows the image which corresponds to 3 s after the flash pulse. The ballistic impact damage is clearly differentiated from sound areas in the raw image shown in Figure 4b. Two points, one on the impact area and another on a sound area (red and blue, respectively), are plotted on these images. Temperature profiles of these two points are shown in Figure 4d. MWIR and LWIR raw sequences were used to train and test two different deep learning models for impact damage segmentation (one spectral band for each model).

PT is probably the most extensively investigated approach because it is fast (from a few seconds for high conductivity materials to several seconds for low conductivity materials) and easy to deploy. Raw PT data, however, is difficult to handle and analyze. Therefore, some damage could not be identified if one analyzes only the raw IR sequence. There are a great variety of processing techniques available to enhance the subtle IR signatures.

A very simple approach is a thermal contrast technique. Thermal contrast is a basic operation that despite its simplicity is at the origin of most of the PT analysis. Various thermal contrast definitions exist [14], but they all share the need to specify a sound area $S_{a}$: a non-defective region within the field of view. The absolute thermal contrast Δ(t):

(1) $$\Delta \left(t\right)=T_{d}\left(t\right)-T_{Sa}\left(t\right)$$

where T(t) is the temperature at time *t*, $T_{d}\left(t\right)$ is the temperature of a single pixel or the average of a group of pixels, and $T_{Sa}\left(t\right)$ is the temperature at time *t* for a sound area. No defect can be detected at a particular time *t* if T(t)=0.

Another processing technique proposed in [23] is called principal component thermography (PCT). It relies on singular value decomposition (SVD), which is a tool to extract spatial and temporal data from a matrix in a compact manner by projecting original data onto a system of orthogonal components known as empirical orthogonal functions (EOF). By sorting the principal components in such a way that the first EOF represents the most characteristic variability of the data, the second EOF contains the second most important variability, and so on.

The SVD of a MxN matrix *A*, where M>N, can be calculated as follows:

(2) $$A=URV^{T}$$

where *U* is a M×N matrix, *R* is a diagonal N×N matrix (with singular values of *A* present in the diagonal), and $V^{T}$ is the transpose of a N×N orthogonal matrix (characteristic time) as proposed in [23].

Hence, in order to apply the SVD to thermographic data, the 3D thermogram matrix representing time and spatial variations has to be reorganised as a 2D M×N matrix *A*. This can be done by rearranging the thermograms for every time as columns in *A*, in such a way that time variations will occur column-wise while spatial variations will occur row-wise. Under this configuration, the columns of *U* represent a set of orthogonal statistical modes known as EOF that describe the data spatial variations. On the other hand, the principal components (PCs), which represent time variations, are arranged row-wise in matrix $V^{T}$. The first EOF will represent the most characteristic variability of the data, the second EOF will contain the second most important variability, and so on. Usually, original data can be adequately represented with only a few EOFs. Typically, an IR sequence of 1000 images can be replaced by 10 or less EOFs.

**Figure 5:**
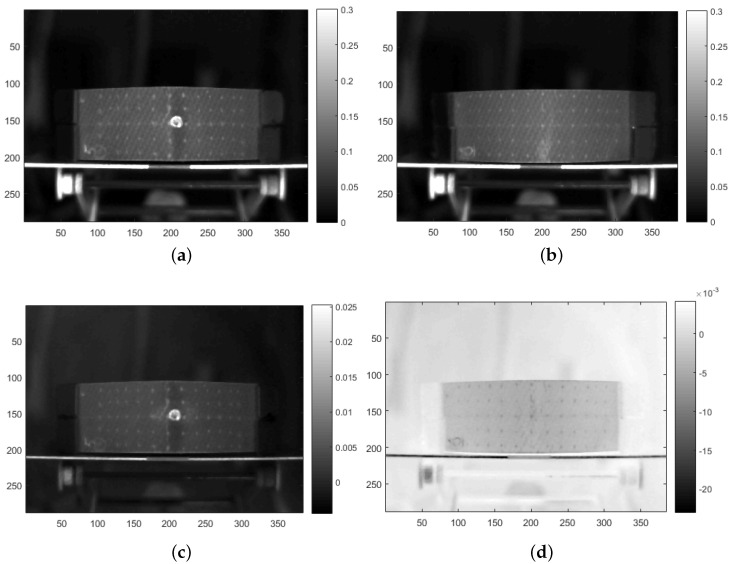
PCT second components obtained from: MWIR sequences, (**a**) specimen 5 (impacted) and (**b**) specimen 6 (non-impacted); and LWIR sequences, (**c**) specimen 5 (impacted) and (**d**) specimen 6 (non-impacted).

In this study, to correctly label the data for training, PCT was applied for defect visualization enhancement. Figure 5 shows the second components obtained for specimens 5 (impacted) and 6 (non-impacted) on MWIR data. Even though the first EOF brings the most characteristic variability of the data, it does not brought useful information for damage detection. Therefore, second and third EOFs were used. In general, for PT, the first EOF is affected by the flash pulse being slightly saturated. Thus, second and third EOFs are usually used for the purpose of damage detection. In Figure 5b we can confirm that there was no damage in the sound specimen, as was expected. The entire extent of the impact damage is clearly visible in Figure 5a. Results shown in Figure 5a are from the sequence of images shown in Figure 5c. Figure 5c,d shows results for the same specimen but with images from the LWIR data sequence. Impact damage defect shape and position were considered from these images, i.e., PCT results, for network labeling target classes. Other defects are visible in the PCT images, such as cracks and delaminations. In this work, the goal was to segment only the impact damage region.

### 2.4. Artificial Intelligence Tools Applied in Infrared Thermography

In the last decade, several groups studied the use of artificial intelligence tools for NDT&E. Oliveira et al. presented a transfer learning case in [24] with the application of a U-Net convolutional neural network, which was optimised for processing medical images, for segmenting impact damages in infrared phase images of carbon fibre reinforced plastic plates, which were acquired using optical lock-in thermography. Bang et al. [25] proposed a framework for identifying defects in composite materials by integrating a thermography test with a deep learning technique. A dataset of thermographic images of composite materials with defects were collected from literature and were used for training the system to identify defects from given thermographic images. The identification algorithm adopts faster region based convolutional neural network (RCNN) for identifying an object, i.e., defect(s) in this case, by employing an automatic learning feature from the available data. In [26], Marani et al. developed a complete approach for the automatic detection and classification of defects in composite materials for aeronautics. They used a decision forest made of 30 trees trained on the input image; it was able to classify three classes of surface areas (pristine, surface defects, and internal defects) with high values of both standard accuracy and balanced accuracy.

#### Convolutional Neural Networks

Convolutional neural networks (CNNs), as an important area of deep learning, have leveraged tremendous improvements in image classification [27,28,29] and image segmentation [30,31,32]. However, their applications in infrared images have not been well explored yet. Unlike visible detectable images, which can be easily obtained, infrared data are employed to characterize objects through very expensive equipment. Moreover, CNNs contain a large number of parameters and have strong ability for non-linear predication. Therefore, this approach demands plenty of training data samples to facilitate good generalization. There are several approaches available in the literature for image segmentation and image classification. Some of them are briefly described next.

ResNet: It stands for deep residual network [29]. As the name indicates, it introduces what is called residual learning. In general, in deep CNNs several layers are stacked and trained to learn features. In residual learning, instead of learning features, the network learns residues. The residue is actually a subtraction of the input of the current layer from the output of this layer. ResNet learns residues using shortcut connections that link the *n*th layer to the n+1th layer. Literature results have shown that it is easier to train ResNet than classical CNNs. ResNet can be configured up to over 1000 layers.

DenseNet: It is a logical extension of ResNet having as a fundamental building block the concept of residue connections. In contrast with ResNet, DenseNet proposes to concatenate the previous layers instead of using a summation. DenseNet [33] connects each layer to every other layer in a feed-forward fashion. While traditional CNN with *L* layers have L groups of connections—a group between each layer and its subsequent layer—DenseNet has L(L+1)/2 groups of connections. In summary, for each layer, feature maps of all predecessor layers are used as input, and its output map is used as input to all subsequent layers. DenseNet used can be considered a small CNN having 8 millions of parameters.

PSPNet: One of the strategies used to improve the performance of semantic segmentation is the use of context clues [34]. However, the primary issue for current fully convolutional networks (FCN) based models is the lack of a suitable strategy to use global scene category clues [35]. It is essential to the defects of the context because of the features around. In this way, we were motivated to use an architecture based on spatial pyramidal pooling such as the PSPNet network [35]. Given an input image, PSPNet uses a pre-trained ResNet [29] with the dilated network strategy [36] to extract the feature map. The final feature map size is 1/8 of the input image. Applying the 4-level pyramid, the pooling kernels cover the image. They are combined as the global, concatenate the previous with the original feature map, accompanied by a convolution layer to produce the final prediction map.

U-Net: There is consensus that successful training of a CNN requires many thousands of training samples. However, getting thousands of training images of the defects labeled is not a simple task. We need architectures that require few training images and provide good results. In this case, U-Net [30] is highlighted because it introduces skip connections between the downsampling step and the upsampling step, which allows the latter step to use the features from earlier steps. During U-Net’s training, it uses strategies like data augmentation to apply the labeled images efficiently. As a result, it provides superior performance when segmenting the medical images.

### 2.5. Proposed Approach

#### 2.5.1. Network Architecture

**Figure 6:**
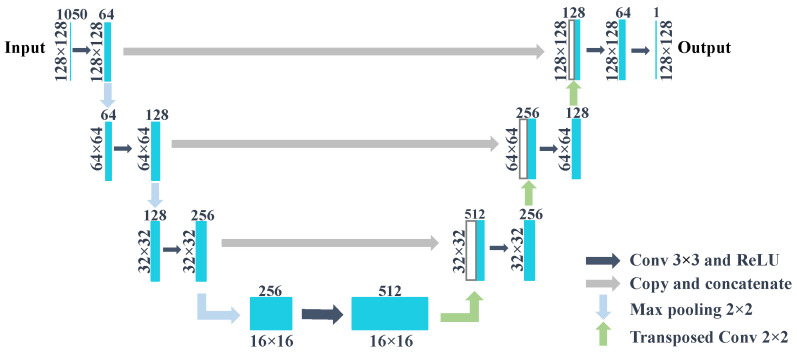
U-Net architecture.

The network architecture used in this work is shown in Figure 6. The network consists of eight steps: the first three steps are downsampling steps; the fourth step is located in the bottom of the net is named bottom step; the fifth to seventh steps are three upsampling steps; and the last step is to output the two-dimensional heat map for prediction. For the upsampling and downsampling steps, each of them contains a data processing chain: at first a convolutional layer; then a batch normalization, a rectified linear unit layer (ReLU), and a 2 × 2 max pooling layer with a stride of two; and after that a max pooling layer for the downsampling step and transposed convolution for the upsampling step. The bottom step contains a convolutional layer, a batch normalization layer, and ReLU, and it does not have max pooling layer or a transposed convolution layer. The last step only has a convolutional layer. Unlike the original U-Net that contains two convolutional layers in each step, here only one convolutional layer was used in each step, because with this setting the network generalized better on the validation dataset. This network takes 128 × 128 × 1050 image patches as inputs and generates a 128 × 128 image as output.

#### 2.5.2. Experiment

In this section, the details of the experiment will be discussed, including five steps: data preparation, data labeling, model training, thresholding, and evaluation.

Data preparation: There were in total eight specimens, of which four specimens contained a damage spot caused by ballistic impact, and four measurements were considered to be damage-free, i.e., non-impacted specimens. For each specimen, there were two distinct measurements: LWIR and MWIR. Since no temperature analysis was performed (pixel numerical value provided by the camera is used), and conditions in the experimental room were controlled, environmental effects on the data may be neglected. i.e., environmental factors did not negatively affect the models, since training, validation, and test sets were all acquired under the same conditions. Each measurement (MWIR and LWIR) is in this case unique. Considering that if they are split into different groups, the training data may not be able to represent the data effectively since there are few specimens available, each measurement was at first cropped with the center point into two parts, left and right, to double the number of available specimens. The exact data splitting is listed in Table 4 along with the training–validation–testing division (75%–12.5%–12.5%).

**Figure 7:**
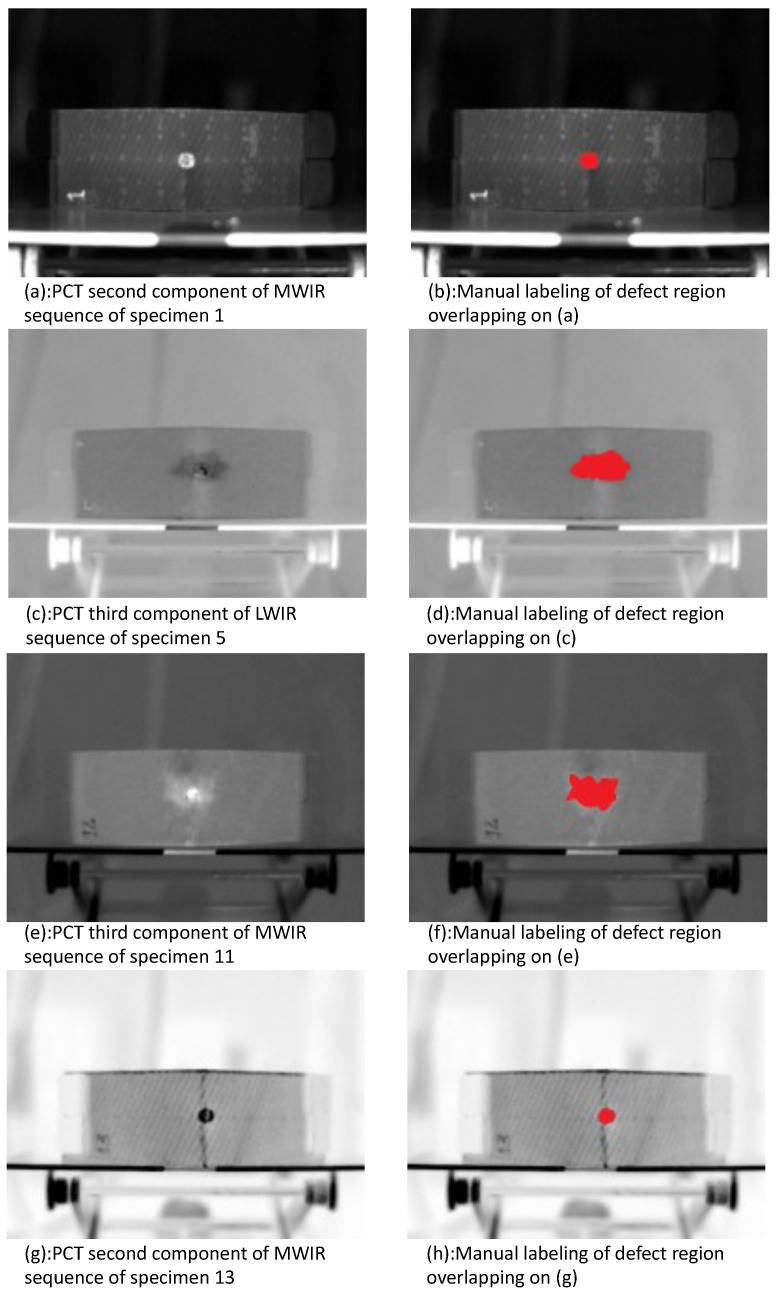
PCT results of impacted specimen and corresponding labeling.

Data labeling: In this case the problem is addressed with a supervised learning approach, where the data firstly need to be labeled. All infrared sequences, in both middle and long range spectra, were processed with PCT [23]. The generated images were then further analyzed, and the components that exhibited better damage visualization for each specimen were chosen. The damage regions (for impacted specimens) were then manually identified and marked at the pixel level. The labeling was done with commercially available image editor software. First, the boundaries of the damaged regions were carefully marked and the region inside could be then filled with red. In this case, as there were only a few regions to be labeled; it did not take long to finish the manual labeling. However, there are some software tools available for labeling that could help accelerate the labeling process when it comes to a large number of images to be labeled [37,38]. Further, for each sequence there will be a two-dimensional binary ground truth image, where the 1s denote damaged regions and the 0s assign damage-free regions. The labels for each damaged region are shown for the four inspected specimens in Figure 7.

Model training: The input data size is 128 × 128 × 1050, and the size of each measurement is 288 × 384 × 1050. Each input data sequence is randomly cropped from the measurement, and is then rotated with a random angle ranging from 0${}^{\circ }$ to 90${}^{\circ }$. The empty border areas are inserted with zero values. After that, each input data sequence is normalized with the following equation to guarantee that the mean value is zero, and the standard deviation is 1.

(3) $$y_{i,j,k}=\frac{x_{i,j,k}-\mu }{\sigma },$$

where $x_{i,j,k}$ is the value of a voxel on one 3D input patch; μ and σ are the mean value and standard deviation of each 3D input patch, respectively.

**Figure 8:**
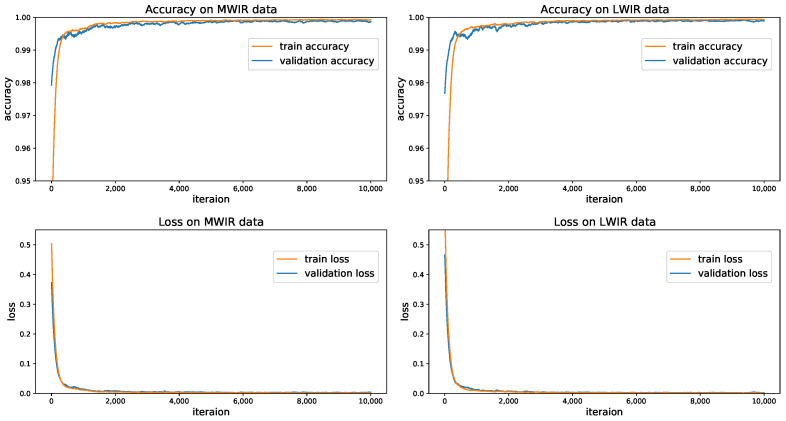
Learning curves during the training process.

Since the MWIR and LWIR data for each specimen were known, a deep learning model was trained for each wavelength. Both models were trained with back-propagation with a learning rate of 0.002, and were trained for 10,000 iterations. The optimizer used here was Adam [39]. The experiment was performed on a GTX 1080 Ti, and the training process lasted about two days. The smoothed learning curves for both models were shown in Figure 8.

Thresholding: The output of the U-Net model is a two-dimensional heat map, where each pixel has a value ranging from 0 to 1 denoting the possibility degree for the damage appearance. Before the map is compared to the ground truth image, it needs to be binarized to get a two-dimensional binary mask. This threshold for the binarization was chosen as 0.5.

Evaluation: After the thresholding process, for each measurement there will be a two-dimensional binary image available. This binary image can be compare to the corresponding ground truth image pixel by pixel for evaluation. The pixels belonging to the damage region are the positive elements to be detected. Then, accuracy, recall, precision, and F1-score for each measurement processed with deep model can be easily obtained.

### 2.6. Model Explainability

Deep learning methods have drawn a lot of attention from the industrial area. The wide scale of deployments in industry, for example, has not yet been realized. One of the main reasons is that in real applications, a rare case that the model has never seen before might happen, and in this case the model’s behavior might be unpredictable. Another important reason is about the deep model’s low level of transparency and explainability. It is hard for end user to fully trust a machine that makes decision based on a black box. However, the deep model’s lack of explainability is due to its complexity. For example, a typical CNN model contains multiple convolutional layers, and each layer is composed of dozens of filters and non-linear activation functions. It is challenging to figure out every detail in the inference process.

**Figure 9:**
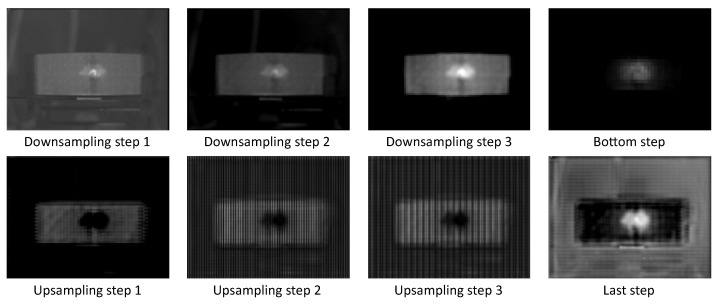
Layer activation maps of a trained deep model for MWIR data.

**Figure 10:**
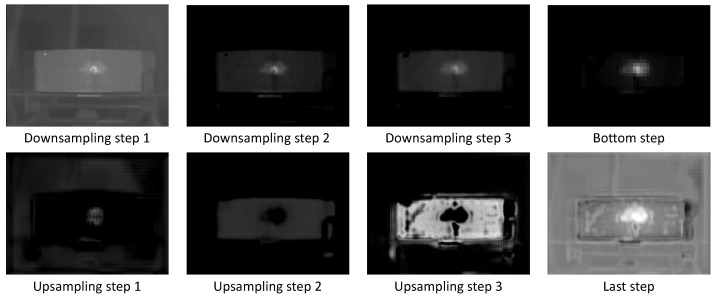
Layer activation maps of a trained deep model for LWIR data.

Due to its importance, this area has been quite actively researched in recent years [40,41,42,43]. There are several approaches addressing this problem: visualizing input patches that maximize a output unit activation [44]; the input attribution method to highlight certain input data areas that contribute the most to a model’s decision [40,45]. In this case the layer activation method implemented in the framework Captum [46] for Explainable AI is applied on the trained model. The layer activation method is simply to compute the activation of each neuron in the identified layer. With this method, layer activation at the end of each step in the U-Net is calculated and visualized. As Figure 9 and Figure 10 demonstrate, the model tends to generate hot spots on the damaged regions. In this way, the inner functionality can be revealed to a certain extent.

Several activation maps are shown in Figure 9 and Figure 10. There are eight steps in total over the network, each of which comprises convolutional layer, max pooling or transposed convolutional layer, and a ReLU as activation function. For each step, one of the activation maps was selected and enhanced for visualization. The eight activation maps were able to provide an overview of the data process during both networks’ inferences (the network for MWIR data and the network for LWIR data). The input data for both networks were gathered from specimen 5. The data indicate that after the first step (downsampling step 1), the input image was only seeming to be enhanced, and afterwards the network tended to highlight the central damage spot. Right after the bottom step, the central white spot is the only visible object on the image, which means that the network has already detected the damage location in early steps, and the following steps could be possibly used only to predict the exact boundary of the damage spot. Additionally, the last step output, i.e., the final output of the network, is a reconstructed two-dimensional heat map which describes both the component outline and the damage location.

## 3. Results

The evaluation was performed on two specimens’ parts: left part of specimen 6 and right part of specimen 13 (“Specimen 06 left” and “Specimen 13 right” in Table 4 respectively, which were randomly chosen to be in the test set). Results for the model trained with the MWIR sequences are presented in Table 5 and results for the model trained with the LWIR sequences are presented in Table 6. “Specimen 13 right” contains a damage region, while “Specimen 06 left” is damage-free. Accuracy, recall, precision, and F1-score were chosen as evaluation measures. Since the damaged area was too small compared to the whole image, accuracy alone is not the best measure to evaluate the results. Even an accuracy of 99% may not mean an actual high performance. F1-score is a better choice to rank the overall performance of the deep learning model. Since the left part of specimen 6 contains no damage, the recall, precision, and F1-Score are not available. However, the classifier achieved 100%. Thus, in this case, it is feasible to use only accuracy to measure the model’s performance.

## 4. Discussion

### 4.1. PCT Analysis

Following what was described in Section 2, PT was used to inspect the specimens listed in Table 3. Acquired data were used to train to DL models to segment impact damages from raw PT data. However, to train and test the models, target regions, i.e., ground truth images, should be known. In this study, PCT, a method based on SVD, was applied in the raw sequences in order to gain knowledge on the damages present in the specimens and further label damage regions. Thus, PCT was a intermediary step on our approach. Nevertheless, it was useful for understanding the extent of the damages caused by the ballistic impact in the curved specimens.

For example, specimens 5 and 11, which were submitted to more thermal shock cycles before the ballistic impact, 300 and 500 respectively, presented a higher amount of damage, as can be seen in Figure 7c,e. In addition to the impact damage, PCT results for these specimens presented cracks and delamination (butterfly shape around the impacted region). On the other hand, specimens 1 and 13, which were submitted to fewer thermal shock cycles before the ballistic impact, 150 and 0 respectively, as shown in Figure 7a,g, did not present the same degree of damage as specimens 5 and 11. This clearly indicates that the thermal shocks stressed the specimens, which made them more susceptible to the ballistic impact.

### 4.2. Testing Results of Deep Models

The outputs of both networks are two-dimensional heat maps. Each location of the map contains a value ranging from 0 to 1 indicating the likelihood of a pixel belongs to a damaged area. The heat map is binarized with a default threshold of 0.5. The threshold is a hyper-parameter and can be tuned before the evaluation process. Experiments indicate that with a fine-tuned threshold value, the evaluation results can be slightly improved. However, the fine-tuned threshold value is highly sensitive to the training epochs. and therefore is not representative. Therefore, the center value 0.5 of the output value range [0, 1] was chosen here in consideration of reproducibility of the work. The reason for the sensitivity could be the lack of training samples, which would hinder the model’s generalization.

On the one hand, both deep models for MWIR data and LWIR data can recognize the absolute damage-free area with an accuracy of 100% (on “Specimen 06 left”) with a default threshold of 0.5. On the other hand, for the specimen containing a defect spot, both above-mentioned models were not able to generate comparable results. The models for MWIR data and LWIR data reached F1-scores of 92.74% and 87.39%, respectively.

**Figure 11:**
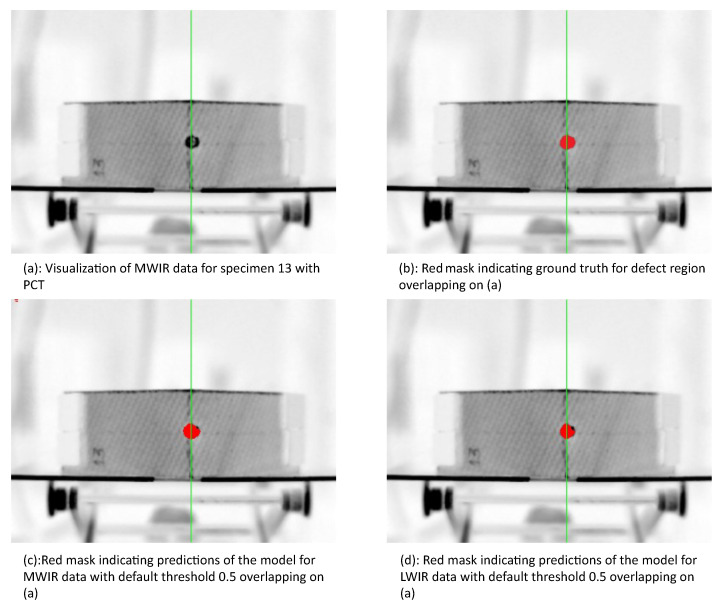
Visualization of results from specimen 13: The specimen was split into left and right parts; the left part was considered training data, and the right part was considered testing data. The green line denotes the splitting boundary.

However, the results are still satisfactory for the damage detection task. As is shown in Figure 11, both models predicted the location of the damage correctly with the default threshold. Some false positives, i.e., sound pixels classified as damage, were only detected at the border area. The reasons could be that the sound region close to the damage region suffered from some thermal influence by the damage during the IRT experiment, and the manual labeling process is not 100% accurate.

### 4.3. Model Explainability

Though the model with U-Net architecture is able to recognize the damaged region well, its transparency is still low, not only because of the convolutional layers containing plenty of parameters but also because of the skip connections between the downsampling steps and upsampling steps that have increased the complexity of the model. In this case, the input attribution is not suitable, as it is quite intuitive that the area around the damaged region should contribute the most to the model’s prediction. Therefore, the layer activation method was used here to visualize the output of the each step, so that one can see how the data are processed by the deep models. Nevertheless, this method still has some limitations, since the explanation is in this case also quite dependent on the input data, and in some middle steps there are up to 512 activation maps available, so it is probably not feasible to carefully analyze each of them one by one.

## 5. Conclusions

NDT&E of CFRP with infrared thermography incorporating deep learning is presented in this paper. IRT was used to inspect curved specimens in both MWIR and LWIR spectra, and IR data were processed with PCT to acquire ground truth images used for model training. Two deep models with U-Net architecture were trained to predict the damage region on MWIR and LWIR, respectively. For the damage-free specimen, the model reached high accuracy (in this case 100%). On the damaged specimen, both models were able to correctly identify the damage. Besides, both models can also predict the damaged region’s location with an F1-score of 92.74% on MWIR data and an F1-score of 87.39% on LWIR.

Since the model trained on MWIR data only outperformed the model trained on LWIR data by a limited margin, both testing methods would contain sufficient latent information to allow accurate damage detection. It is worth mentioning that during PCT analysis, MWIR images showed better details when compared to LWIR data. It is possible to observe in Figure 5 that the results obtained from the MWIR sequence for specimen 5 and 6 displayed clearer results. e.g., in Figure 5a we can see sharper boundaries around the impact damage region. This implies that the latent information on LWIR data may not be well represented by the PCT approach. Therefore, the deep learning method with U-Net was shown to ve superior in unveiling latent relevant information in IR data compared to the heuristic PCT method, since the F1-scores of both models are close.

After the evaluation, both models were analyzed with the layer activation method to explain the data processing mechanisms. The layer activation map at the end of each step was computed and visualized. Those visualized activation maps showed that both models roughly found the damage spot location in the middle steps, whereas the exact region of the damage spot was estimated in the last few steps. This provides an overview of what is happening during the model’s inference, thereby improving the transparency of both deep models with U-Net architecture. In addition, the greater damage extent revealed by PCT in the specimens submitted to higher thermal shock cycles will be further analyzed and the deep learning models described here will be extended to include segmentation of other kinds of damage and their classification.

## Figures and Tables

**Table 1:** Basic physical properties of neat resin.

Property	Value
Specific gravity	$1.35\;g/cm^{3}$
Tg (glass transition)	$90\;{}^{\circ }C$
Tm (melting)	$280\;{}^{\circ }C$
Tp (processing)	300–$330\;{}^{\circ }C$

**Table 2:** Basic physical properties of individual pre-impregnated ply.

Property	Value
Fibre areal weight	$280\;g/m^{2}$
Weight per ply	$496\;g/m^{2}$
Resin content by weight	43%
Consolidated ply thickness	0.31 mm
Density	$1.55\;g/cm^{3}$
Width	1270 mm

**Table 3:** Characteristics of curved specimens inspected by IRT (y: yes, n: no).

Specimen	Thermal Shock Cycles	Impacted (y/n)
1	150	y
2	150	n
5	300	y
6	300	n
11	500	y
12	500	n
13	0	y
15	0	n

**Table 4:** Data splitting for LWIR and MWIR.

Training Data	Validation Data	Test Data
Specimen01 left	Specimen02 right	Specimen06 left
Specimen01 right	Specimen05 left	Specimen13 right
Specimen02 left		
Specimen05 right		
Specimen06 right		
Specimen11 left		
Specimen11 right		
Specimen12 left		
Specimen12 right		
Specimen13 left		
Specimen15 left		
Specimen15 right		

**Table 5:** Deep model’s evaluation results on MWIR testing data.

	Specimen 13 Right Part	Specimen 06 Left Part
Threshold	0.5	0.5
Accuracy	99.96%	100.00%
Recall	93.50 %	/
Precision	92.00%	/
F1-score	92.74%	/

**Table 6:** Deep model’s evaluation results on LWIR testing data.

	Specimen 13 Right Part	Specimen 06 Left Part
Threshold	0.5	0.5
Accuracy	99.94%	100.00%
Recall	78.86%	/
Precision	97.98%	/
F1-score	87.39%	/

## Data Availability

All data generated or appeared in this study are available upon request by contact with the corresponding author.

## Abbreviations

The following abbreviations are used in this manuscript:

AI	Artificial intelligence
CM	Composite materials
CNN	Convolutional neural network
EOF	Empirical orthogonal function
FPS	Frames per second
IR	Infrared
IRT	Infrared thermography
LEO	Low earth orbit
LWIR	Long-wave infrared
MWIR	Mid-wave infrared
NASA	National Aeronautics and Space Administration
NDT&E	Non-destructive testing and evaluation
PCT	Principal component thermography
PT	Pulsed thermography
PSS	Polyphenylene sulfide
PT	Pulsed thermography
ReLU	Rectified linear unit layer
SMAP	Soil Moisture Active Passive
SVD	Singular value decomposition

## References

[1] McIlhagger A., Archer E., McIlhagger R., Irving P., Soutis C. (2015). Manufacturing processes for composite materials and components for aerospace applications. Polymer Composites in the Aerospace Industry.

[2] Timmis A.J., Hodzic A., Koh L., Bonner M., Soutis C., Schäfer A.W., Dray L. (2015). Environmental impact assessment of aviation emission reduction through the implementation of composite materials. Int. J. Life Cycle Assess..

[3] Summa J., Becker M., Grossmann F., Pohl M., Stommel M., Herrmann H.G. (2018). Fracture analysis of a metal to CFRP hybrid with thermoplastic interlayers for interfacial stress relaxation using in situ thermography. Compos. Struct..

[4] Yang B., Yue Z., Geng X., Wang P., Gan J., Liao B. (2018). Effects of space environment temperature on the mechanical properties of carbon fiber/bismaleimide composites laminates. Proc. Inst. Mech. Eng. Part G J. Aerosp. Eng..

[5] Mahdavi S., Gupta S.K., Hojjati M. (2018). Thermal cycling of composite laminates made of out-of-autoclave materials. Sci. Eng. Compos. Mater..

[6] Gupta S.K., Hojjati M. (2019). Microcrack Detection in Composite Laminates at Early Stage of Thermal Cycling Using Moisture/Freeze/Dry Cycle. Int. J. Compos. Mater..

[7] Zhang H., Yu L., Hassler U., Fernandes H., Genest M., Robitaille F., Joncas S., Holub W., Sheng Y., Maldague X. (2016). An experimental and analytical study of micro-laser line thermography on micro-sized flaws in stitched carbon fiber reinforced polymer composites. Compos. Sci. Technol..

[8] Berger D., Brabandt D., Bakir C., Hornung T., Lanza G., Summa J., Schwarz M., Herrmann H.G., Pohl M., Stommel M. (2017). Effects of defects in series production of hybrid CFRP lightweight components–detection and evaluation of quality critical characteristics. Measurement.

[9] Kelkel B., Popow V., Gurka M. (2020). Inline quantification and localization of transverse matrix cracking in cross-ply CFRP during quasi-static tensile testing by a joint event-based evaluation of acoustic emission and passive IR thermography. Compos. Sci. Technol..

[10] Yousefi B., Sfarra S., Sarasini F., Ibarra-Castanedo C., Maldague X. (2019). Low-rank sparse principal component thermography (sparse-PCT): Comparative assessment on detection of subsurface defects. Infrared Phys. Technol..

[11] Schwarz M., Schwarz M., Herter S., Herrmann H.G. (2019). Nondestructive Testing of a Complex Aluminium-CFRP Hybrid Structure with EMAT and Thermography. J. Nondestruct. Eval..

[12] Dilonardo E., Nacucchi M., De Pascalis F., Zarrelli M., Giannini C. (2020). High resolution X-ray computed tomography: A versatile non-destructive tool to characterize CFRP-based aircraft composite elements. Compos. Sci. Technol..

[13] Zhang H., Sfarra S., Sarasini F., Santulli C., Fernandes H., Avdelidis N., Ibarra-Castanedo C., Maldague X. (2018). Thermographic Non-Destructive Evaluation for Natural Fiber-Reinforced Composite Laminates. Appl. Sci..

[14] Maldague X. (2001). Theory and Practice of Infrared Technology for Nondestructive Testing.

[15] Laureti S., Malekmohammadi H., Rizwan M.K., Burrascano P., Sfarra S., Mostacci M., Ricci M. (2019). Looking Through Paintings by Combining Hyper-Spectral Imaging and Pulse-Compression Thermography. Sensors.

[16] Fernandes H., Zhang H., Figueiredo A., Malheiros F., Ignacio L.H., Sfarra S., Ibarra-Castanedo C., Guimaraes G., Maldague X. (2018). Machine Learning and Infrared Thermography for Fiber Orientation Assessment on Randomly-Oriented Strands Parts. Sensors.

[17] Xu C., Xie J., Wu C., Gao L., Chen G., Song G. (2018). Enhancing the Visibility of Delamination during Pulsed Thermography of Carbon Fiber-Reinforced Plates Using a Stacked Autoencoder. Sensors.

[18] Hu C., Duan Y., Liu S., Yan Y., Tao N., Osman A., Ibarra-Castanedo C., Sfarra S., Chen D., Zhang C. (2019). LSTM-RNN-based defect classification in honeycomb structures using infrared thermography. Infrared Phys. Technol..

[19] Duan Y., Liu S., Hu C., Hu J., Zhang H., Yan Y., Tao N., Zhang C., Maldague X., Fang Q. (2019). Automated defect classification in infrared thermography based on a neural network. NDT E Int..

[20] Fang Q., Maldague X. (2020). A Method of Defect Depth Estimation for Simulated Infrared Thermography Data with Deep Learning. Appl. Sci..

[21] Motoyashiki Y., Hasegawa S., Okudaira K., Sato E. (2008). Micrometeoroid impact on ceramic thin components for interplanetary probe. Int. J. Impact Eng..

[22] Maldague X., Maldague X., Moore P.O. (2001). Techniques of infrared thermography: Part 2 Pulse Thermography. Nondestructive Handbook, Infrared and Thermal Testing.

[23] Rajic N. (2002). Principal component thermography for flaw contrast enhancement and flaw depth characterisation in composite structures. Compos. Struct..

[24] Oliveira B.C.F., Seibert A.A., Borges V.K., Albertazzi A., Schmitt R.H. (2020). Employing a U-net convolutional neural network for segmenting impact damages in optical lock-in thermography images of CFRP plates. Nondestruct. Test. Eval..

[25] Bang H.T., Park S., Jeon H. (2020). Defect identification in composite materials via thermography and deep learning techniques. Compos. Struct..

[26] Marani R., Palumbo D., Renò V., Galietti U., Stella E., D’Orazio T. (2018). Modeling and classification of defects in CFRP laminates by thermal non-destructive testing. Compos. Part B Eng..

[27] Krizhevsky A., Sutskever I., Hinton G.E., Bartlett P. (2012). Imagenet classification with deep convolutional neural networks. Advances in Neural Information Processing Systems.

[28] Kim Y. Convolutional Neural Networks for Sentence Classification. Proceedings of the 2014 Conference on Empirical Methods in Natural Language Processing (EMNLP).

[29] He K., Zhang X., Ren S., Sun J. (2015). Deep Residual Learning for Image Recognition. CoRR.

[30] Ronneberger O., Fischer P., Brox T. (2015). U-net: Convolutional networks for biomedical image segmentation. Medical Image Computing and Computer-Assisted Intervention—MICCAI 2015, Proceedings of the International Conference on Medical Image Computing and Computer-Assisted Intervention, Munich, Germany, 5–9 October 2015.

[31] Badrinarayanan V., Kendall A., Cipolla R. (2017). Segnet: A deep convolutional encoder-decoder architecture for image segmentation. IEEE Trans. Pattern Anal. Mach. Intell..

[32] Bhowmick S., Satish Nagarajaiah A.V. (2020). Vision and Deep Learning-Based Algorithms to Detect and Quantify Cracks on Concrete Surfaces from UAV Videos. Sensors.

[33] Huang G., Liu Z., Van Der Maaten L., Weinberger K.Q. Densely Connected Convolutional Networks. Proceedings of the 2017 IEEE Conference on Computer Vision and Pattern Recognition (CVPR).

[34] Yuan Q., Fang Y., Yang J., Hu Q., Cheng M.M., Wang L., Liu Q., Bai X., Meng D. (2017). A Novel Automatic Grouping Algorithm for Feature Selection. Computer Vision.

[35] Zhao H., Shi J., Qi X., Wang X., Jia J. Pyramid Scene Parsing Network. Proceedings of the 2017 IEEE Conference on Computer Vision and Pattern Recognition (CVPR).

[36] Chen L.C., Papandreou G., Kokkinos I., Murphy K., Yuille A.L. (2016). Semantic Image Segmentation with Deep Convolutional Nets and Fully Connected CRFs. arXiv.

[37] Wada K. (2016). Labelme: Image Polygonal Annotation with Python. https://github.com/wkentaro/labelme.

[38] Gupta A.K. (2017). Imglab: To Speedup and Simplify Image Labeling/ Annotation Process with Multiple Supported Formats. https://github.com/NaturalIntelligence/imglab.

[39] Kingma D.P., Ba J. (2014). Adam: A Method for Stochastic Optimization. arXiv.

[40] Bach S., Binder A., Montavon G., Klauschen F., Müller K.R., Samek W. (2015). On pixel-wise explanations for non-linear classifier decisions by layer-wise relevance propagation. PLoS ONE.

[41] Yosinski J., Clune J., Nguyen A.M., Fuchs T.J., Lipson H. (2015). Understanding Neural Networks Through Deep Visualization. CoRR.

[42] Simonyan K., Vedaldi A., Zisserman A. (2014). Deep inside convolutional networks: Visualising image classification models and saliency maps. arXiv.

[43] Oh S.J., Schiele B., Fritz M. (2019). Towards reverse-engineering black-box neural networks. Explainable AI: Interpreting, Explaining and Visualizing Deep Learning.

[44] Nguyen A., Yosinski J., Clune J. (2019). Understanding neural networks via feature visualization: A survey. Explainable AI: Interpreting, Explaining and Visualizing Deep Learning.

[45] Zhou B., Khosla A., Lapedriza A., Oliva A., Torralba A. Learning deep features for discriminative localization. Proceedings of the IEEE Conference on Computer Vision and Pattern Recognition.

[46] Kokhlikyan N., Miglani V., Martin M., Wang E., Alsallakh B., Reynolds J., Melnikov A., Kliushkina N., Araya C., Yan S. (2020). Captum: A unified and generic model interpretability library for PyTorch. arXiv.

